# Study effect of probiotics and prebiotics on treatment of OVA-LPS-induced of allergic asthma inflammation and pneumonia by regulating the TLR4/NF-kB signaling pathway

**DOI:** 10.1186/s12967-022-03337-3

**Published:** 2022-03-16

**Authors:** Zhiwei Wu, Entezar Mehrabi Nasab, Poonam Arora, Seyyed Shamsadin Athari

**Affiliations:** 1 General Internal Medicine Ward, Zhengzhou Central Hospital Affiliated To Zhengzhou University, Zhengzhou City, 450007 China; 2 Department of Cardiology, School of Medicine, Tehran Heart Center, Tehran University of Medical Sciences, Tehran, Iran; 3 Department of Pharmacognosy and Phytochemistry, SGT College of Pharmacy, SGT University, Gurugram, Haryana India; 4 Department of Immunology, School of Medicine, Zanjan University of Medical Sciences, Zanjan, Iran

**Keywords:** Allergy, Microbe, Diet, Immunomodulation, Asthma

## Abstract

Asthma is a common respiratory disease, and immune system dysregulation has direct relevance to asthma pathogenesis. Probiotics and prebiotics have immunomodulatory effects and can regulate immune responses and may attenuate allergic reactions. Therefore, in this study, we explored the role of probiotics and prebiotics in regulating acute airway inflammation and the TLR4/NF-kB pathway. Allergic asthma model of BALB/c mice was produced and treated with probiotics (LA-5, GG, and BB-12) and prebiotics (FOS and GOS). Then AHR, BALF cells count, EPO activity, IL-4, 5, 13, 17, 25, 33, as well as IFN-γ, total and OVA-specific IgE, IgG1, Cys-LT, LTB4, LTC4, and TSLP levels were measured. Also, the GTP/GOT assay was performed and gene expression of Akt, NLR3, NF-kB, PI3K, MyD88, TLR4, CCL11, CCL24, MUC5a, Eotaxin, IL-38, and IL-8 were determined. Finally, lung histopathological features were evaluated. Treatment with probiotics could control AHR, eosinophil infiltration to the BALF and reduce the levels of immunoglobulins, IL-17, GTP and also decrease mucus secretion, goblet cell hyperplasia, peribronchial and perivascular inflammation and also, EPO activity. It could reduce gene expression of TLR4 and CCL11. On the other hand, IL-38 gene expression was increased by both probiotic and prebiotic treatment. Treatment with probiotics and prebiotics could control levels of IL-4, 5, 13, 25, 33, leukotrienes, the gene expression of AKT, NLR3, NF-κB, MyD88, MUC5a. The prebiotic treatment could control peribronchial inflammation and PI3K gene expression. Both of the treatments had no significant effect on the GOT, TSLP and IL-8, eotaxin and CCL24 gene expression. Probiotics and prebiotics could induce tolerance in allegro-inflammatory reactions and alter immune responses in allergic conditions. Probiotics could also modulate cellular and humoral immune responses and prevent allergic disorders.

## Main findings of the study

- Probiotics controls AHR, eosinophil infiltration to the perivascular and BALF, levels of immunoglobulins, IL-17, GTP and also mucus secretion, goblet cell hyperplasia, EPO activity. It could reduce gene expression of TLR4 and CCL11,
- Probiotics and prebiotics control levels of cytokines (IL-4, 5, 13, 25, and 33), leukotrienes, the gene expression of AKT, NLR3, NF-κB, MyD88, MUC5a, peribronchial inflammation and increase IL-38 gene expression.
- Prebiotic controls and PI3K gene expression.

## Introduction

Asthma, pneumonia, and lung injury are common acute respiratory problems in the clinic and allergic asthma is a relatively prevalent condition worldwide. Patients with asthma cannot be cured completely, and all available treatments generally aim to control the pathological condition. There is a huge economic burden on patients and governments [1, 2]. Allergic asthma is one of the most important health problems, especially in developed countries. Several pathways are involved in the pathophysiology of allergic asthma. Immune system dysregulation is directly related to health and is involved in the pathogenesis of a variety of immune disorders, including allergic diseases. On the other hand, the imbalance of inflammatory and anti-inflammatory factors and also dysregulation of the lung immune system through the TLR4/NF-kB signaling pathway can lead to pulmonary oxidative stress and injury [3, 4].

The therapeutic applications of probiotics and prebiotics in diseases such as asthma and pneumonia were not studied very well. Probiotics and prebiotics have been shown to possess immunomodulatory effects and be involved in the pathophysiology of various diseases. Short-chain (galacto-oligosaccharides) and long-chain (fructo-oligosaccharides or lcFOS) prebiotics along with probiotics (beneficial microbes) have also been noted to prevent allergic sensitization via regulating immune responses. Beneficial probiotic bacteria can modulate immune cells such as Th1, Th2, Th17, Treg, and also B cells [5, 6].

There are many microbial infections can active TLR4 signaling and probiotics as part of commensal gut microbiota, can have effect on the TLRs, especially TLR4 [7, 8]. LPS is structural material of bacteria and TLR4 activation by bacteria is initiated via LPS/TLR4/NF-kB pathway [9, 10]. On the other hand, LPS can induce acute lung injury and inflammation, which leads to accumulation of inflammatory cells in the lung and airway [11, 12]. OVA can induce allergic asthma model but, OVA-LPS can induce asthma and acute inflammation in airways. Also, it can involve with TLR4 and actives TLR4 signaling. Therefore, in this study, we explored the functional mechanisms of probiotics and prebiotics in regulating of the host’s defense against LPS-OVA-induced lung injury and airway inflammation and the TLR4/NF-kB pathway. Also, immuno-inflammatory responses and pulmonary pathological features were studied in the mice that were treated with probiotics and prebiotics. The effect of probiotics and prebiotics of on acute form of allegro-inflammatory airway asthma with TLRs involving was studied.

## Material and methods

### Reagents and chemicals

OVA and LPS were procured from Sigma-Aldrich. Probiotics and prebiotics were obtained from Caruso Natural Health (Sydney, NSW, Australia) and Sebogard Oral, Cantabria Labs Difa Cooper Caronn (Pertusella, Italy), respectively. ELISA kits for total IgE were purchased from BD Biosciences (USA); OVA-specific IgE was prepared from MyBiosource (USA); IgG1 from Abcam (USA); Cys-LT from Cayman Chemical Ann Arbor (USA); LTB4 and LTC4 from MyBiosource (USA); and TSLP from Rndsystems (USA); and cytokines ELISA kits BD Biosciences (USA). All the chemicals and solvents used in various analyses had analytical grades.

### Experimental animals

Total of 90 male BALB/c mice (6–8 weeks) were initially acclimatized to standard animal laboratory conditions for one week prior to experiments. The experiments were strictly conducted in accordance with the protocols approved by the Institutional Animal Ethical Committee (IAEC).

### OVA-LPS sensitization and challenge

For the sensitization, challenge, and treatment protocol, mice were randomly divided into six groups (n = 15/group, five for AHR, five for histopathology, and five for immune-biochemical analyses). Group I was the non-sensitized control group; group II was the OVA + LPS control group, and group III received OVA + LPS + probiotics. Each probiotic oral dose consisted of multi-strain probiotics N25 billion CFU [containing *Lactobacillus acidophilus* LA-5 (7.5 billion), *L. rhamnosus* GG (8.75 billion CFU), and *Bifidobacterium animalis* subspecies lactis BB-12 (8.75 billion CFU)] [13]. Group IV received OVA + LPS + prebiotics [FOS and GOS (10 mg/kg, BW, PO)] in PBS solution. Two treatments were diluted in PBS solution and administrated via oral gavage [14]. Group V was the OVA control group, and group VI was the LPS control group.

For creating asthma models, mice were sensitized on days 1, 7, and 14 by the IP injection of 100 μg OVA emulsified in 1 mg aluminum hydroxide gel in a total volume of 200 μL. On days 15, 17, and 19 after the initial sensitization, the animals of the groups II, III, and IV were challenged IN with OVA + LPS (50 μg of OVA combined with 1 μg of LPS in saline in a total volume of 50 μL) and on days 21, 23, and 25. The mice were challenged for 30 min with 3% OVA aerosol (w/v) in saline. Group V was received OVA and group VI was received LPS following the same protocol. Exposure to the aerosolized solution was done in a closed chamber (40 × 20 × 20 cm). The animals in the healthy control group were exposed to PBS following the same protocol. Treatments with probiotics and prebiotics were done once a day from the day 15 to 25 at four hours after OVA-aerosol inhalation. During the study, all animals received food and water ad libitum. On day 27th after the last OVA challenge, sampling was done.

### Assessment of AHR in response to methacholine challenge

The effects of probiotics and prebiotics on AHR in asthmatic mice were assessed by whole-body plethysmograph and MCh challenge test via intubation. AHR was measured on day 27th after the last challenge by determining the enhanced pause (i.e., the Penh value). Tube of the ventilator was connected to the trachea after anesthesia and surgery. Mice were anesthetized with 1.5% pentobarbital sodium, then tracheotomized, and finally connected to a ventilator. Baseline parameters were determined after exposure to aerosolized PBS for 3 min, followed by exposure to the increasing concentrations of aerosolized MCh (1, 2, 4, 8, 16, and 32 mg/ml in PBS). Each dose was nebulized for 15 min, and airway responses were recorded for 5 min. Following each administration through the inlet of the main chamber, the Penh was recorded for 5 min. The mean Penh values were measured during each 5-min period and for each dose and plotted against changes from the baseline per dose of MCh. Differences in Penh values respective to the baseline at each concentration were used to compare airway reactivity between the experimental groups.

### Collection of BALF and differential cell count

After anaesthetization, 24 h after the last challenge, the mice were euthanized by CO_2_; then tracheotomy was performed. Lungs were lavaged for three times with 1.0 ml aliquots of PBS via a cannulated tracheal tube (1.0 ml × 3). Afterward, BALF was pooled and cryocentrifuged (1500 rpm, 10 min, 4 °C). The supernatant was separated and stored at − 80 °C for immune-biochemical analyses while the cell sediment was used for cell/gene expression studies.

To perform a differential cell count, a total of 2–4 × 10^4^ BALF cells were placed on a slide and centrifuged (750 rpm, 2 min) using a cytospin machine. The slides were dried, and cells were stained using Giemsa. The absolute number of Eos was determined by microscopical analysis.

### EPO activity

EPO activity was determined in BALF. Briefly, 1 ml of a substrate solution containing 0.1 mM O-phenylenediamine dihydrochloride, 0.1%Triton X-100, and 1 mM hydrogen peroxide in 0.05 M Tris (hydroxymethyl) aminomethane hydrochloride was added to 1 ml BALF, and the mixture was incubated at 37 °C for 30 min. The reaction was stopped by adding 0.5 ml 4 M sulfuric acid, and the absorbance was read at 492 nm.

### Analysis of cytokines in BALF

The concentrations of IL-4, -5, -13, -17, -25, -33 and IFN-γ in BALF were measured via specific ELISA kits according to the manufacturer’s instructions.

### Serum immunoglobulins

IgG1, total and OVA-specific IgE levels in serum were measured by ELISA following the manufacturer’s protocol.

### Eicosanoid assessment and biofactors’ levels

In BALF supernatant, Cys-LT, LTB4, LTC4, and TSLP levels were assayed using ELISA kits following the manufacturer’s protocol.

### GTP/GOT assay

Oxidative stress in the lung was examined using a glutathione assay kit (Cayman Chemicals) according to the manufacturer’s instructions.

### Quantitative real-time PCR

RNA from BALF cells was extracted using the RNAiso Plus kit following the manufacturer’s protocol. One µg RNA was reversed transcribed to cDNA in a total volume of 20 µl using the PrimeScript RT Reagent Kit. Target genes’ expressions (Akt, NLR3, NF-kB, PI3K, MyD88, TLR4, CCL11, CCL24, MUC5a, Eotaxin, IL38, and IL8) were studied using SYBR Green Master Mix and specific primers.

The specific primers’ sequences (5ʹ–3ʹ) for RNA amplification were as follows:

GAPDH Forward: TGTTCCTACCCCCAATGTGT, Reverse: GGTCCTCAGTGTAGCCCAAG as housekeeping gene; MUC5a Forward: CAGGACTCTCTGAAATCGTACCA, Reverse: AAGGCTCGTACCACAGGGA;

TLR4 Forward: AGTGGGTCAAGGAACAGAAGCA, Reverse: CTTTACCAGCTCATTTCTCACC; NLR3 Forward: ACCAGCCAGAGTGGAATGA, Reverse: GCGTGTAGCGACTGTTGAG; IL-8 Forward: ACTGAGAGTGATTGAGAGTGGAC, Reverse: AACCCTCTGCACCCAGTTTTC; IL-38 Forward: CCTGGCGTGTGTAAAGACAA, Reverse: CAGATCCCAAGCTTCTCTGG;

CCL24 Forward: AGGCAGTGAGAACCAAGT, Reverse: GCGTCAATACCTATGTCCAA; CCL11: Forward GGCTTCATGTAGTTCCAGAT, Reverse: CCATTGTGTTCCTCAATAATCC; PI3K Forward: CTCTCCTGTGCTGGCTACTGT, Reverse: GCTCTCGGTTGATTCCAAACT; Akt Forward: ATCCCCTCAACAACTTCTCAGT, Reverse: CTTCCGTCCACTCTTCTCTTTC; Eotaxin Forward: CTGCTCACGGTCACTTCCTT, Reverse: GGGGTCAGCACAGATCTCTT; NF-kB Forward: ACCTTTGCTGGAAACACACC, Reverse: ATGGCCTCGGAAGTTTCTTT; MyD88 Forward: TGGCATGCCTCCATCATAGTTAACC, Reverse: GTCAGAAACAACCACCACCATGC.

### Lung histopathological study

Lung tissues were isolated and fixed in formalin; then histological slides were produced and stained with H&E and PAS stains. The slides were evaluated under the light microscopy for detecting eosinophilic infiltration around bronchi and vessels, goblet cell hyperplasia, and mucus hypersecretion. It was performed by two investigators as pathologist, blinded to the status of the animal. The mucus production ratio was determined by visually scoring the intensity of PAS stain (in 10 randomly selected microscopy fields). The score 0 was used for absence of any observable PAS stain and 0. 5 score was used for low level of PAS stain. The score 1 was used when approximately 25% of the space inside of the bronchi was filled with mucus, score 2 fore 50%, score 3 fore 75% and score 4 for 100%. Moreover, number of the goblet cells was quantified per 100 epithelial cells at several randomly microscopy fields on sections at 400 × magnification. The calculated number was GCI. The GCI was classified to 4 scores as follows; GCI < 5%: score 0, 5% ≤ GCI < 25%: score 1, 25% ≤ GCI < 50%: score 2: 50% ≤ GCI < 75%: score 3, 75% ≤ GCI ≤ 100%: score 4. Population of the eosinophils was examined at 1000 × magnification. Absence or presence few eosinophils was scored 0, score between 0.1–1 was used for incomplete layer, one complete layer of eosinophils in peribronchial/perivascular was scored 1, two complete layers was 2, three complete layers was 3, more than three complete layers was scored 4 [2].

### Statistical analysis

One-way analysis of variance (ANOVA) was used to analyze statistical differences between the groups. All data were expressed as mean ± SEM. Statistical analyses were performed and graphs were drawn in GraphPad Prism 5.0 software (GraphPad Software Inc., San Diego, CA, USA). The level of statistical significance was set at p-value < 0.05.

## Results

### Effect of probiotic and prebiotic treatment on AHR

AHR is the most important pathological feature of asthma, which distinguishes it from other airway inflammatory diseases. AHR was assessed by measuring Penh value in OVA-LPS sensitized/challenged mice exposed to the increasing concentrations of inhaled MCh. In our study, AHR significantly increased in the OVA and OVA-LPS sensitized asthma groups compared to healthy control animals (p < 0.05). A higher level of Penh indicated a more severe bronchospasm/AHR to the allergen. However, the oral administration of probiotics and prebiotics to OVA-LPS sensitized/challenged mice improved AHR. The results revealed that from the two different prophylactic interventions, only probiotic administration significantly decreased AHR in asthmatic animals (3 ± 0.7 at the dose 4, and 6 ± 0.2 at the dose 32 mg/ml MCh) as compared to the OVA-LPS sensitized/challenged group (7.5 ± 0.4 at the dose 4, and 14 ± 0.7 at the dose 32 mg/ml MCh), (p < 0.05). Treatment of asthmatic animals with prebiotics also reduced AHR (7 ± 0.4 at the dose 4, and 13.5 ± 0.3 at the dose 32 mg/ml MCh), but the results had not significantly difference compared to the OVA-LPS sensitized/challenged group at the all doses of MCh (p > 0.05).

### Effect of probiotic and prebiotic treatment on BALF cells and EPO activity

The recruitment of activated eosinophils to airways is a characteristic feature of asthma. Eosinophils from patients with allergic asthma have been reported to excessively produce ROS via multiple mechanisms that are known to contribute to airway inflammation and remodeling. In our study, a significant increase in the eosinophil count of the BALF (9 × 10^5^ ± 6 × 10^4^ cells/ml) was observed in OVA-LPS challenged mice (and also OVA challenged group) as compared to normal healthy animals (3 × 10^4^ ± 1.5 × 10^4^ cells/ml) (p < 0.05). We also found that treatment with probiotics markedly decreased the recruitment of eosinophils (6.4 × 10^5^ ± 6 × 10^4^ cells/ml) in the BALF of asthmatic animals (p < 0.05). Prebiotic treatment also decreased eosinophilic infiltration into BALF (7.6 × 10^5^ ± 4.5 × 10^4^ cells/ml), but the result was not statistically significant compared to the non-treated asthma control group. In this study, a significant increase in the neutrophils count of the BALF was observed in groups that were challenged with LPS [LPS (3.7 × 10^5^ ± 3 × 10^4^ cells/ml), OVA-LPS (3.6 × 10^5^ ± 6 × 10^4^ cells/ml), OVA-LPS probiotics (1.6 × 10^5^ ± 3 × 10^4^ cells/ml) and prebiotics (2.2 × 10^5^ ± 4.5 × 10^4^ cells/ml) treated groups] compared to PBS (4.5 × 10^4^ ± 5 × 10^3^ cells/ml) and OVA (6 × 10^4^ ± 5 × 10^3^ cells/ml) groups. Treatment with probiotics and prebiotics could significantly (p < 0.05) control neutrophils infiltration to the BALF.

EPO plays an important role in the pathogenesis of asthma by mediating oxidative events, leading to the production of ROS and RNS. A considerable reduction of EPO was observed in OVA-LPS sensitized mice after oral treatment with probiotics (0.21 ± 0.02) compared to the non-treated asthma group (0.34 ± 0.04). These results implicated that probiotics could have antioxidant properties by reducing EPO activity. Prebiotics (0.3 ± 0.02); however, had no significant (p > 0.05) effects on EPO activity.

### Effect of probiotic and prebiotic treatments on cytokines’ levels

**Fig. 1:**
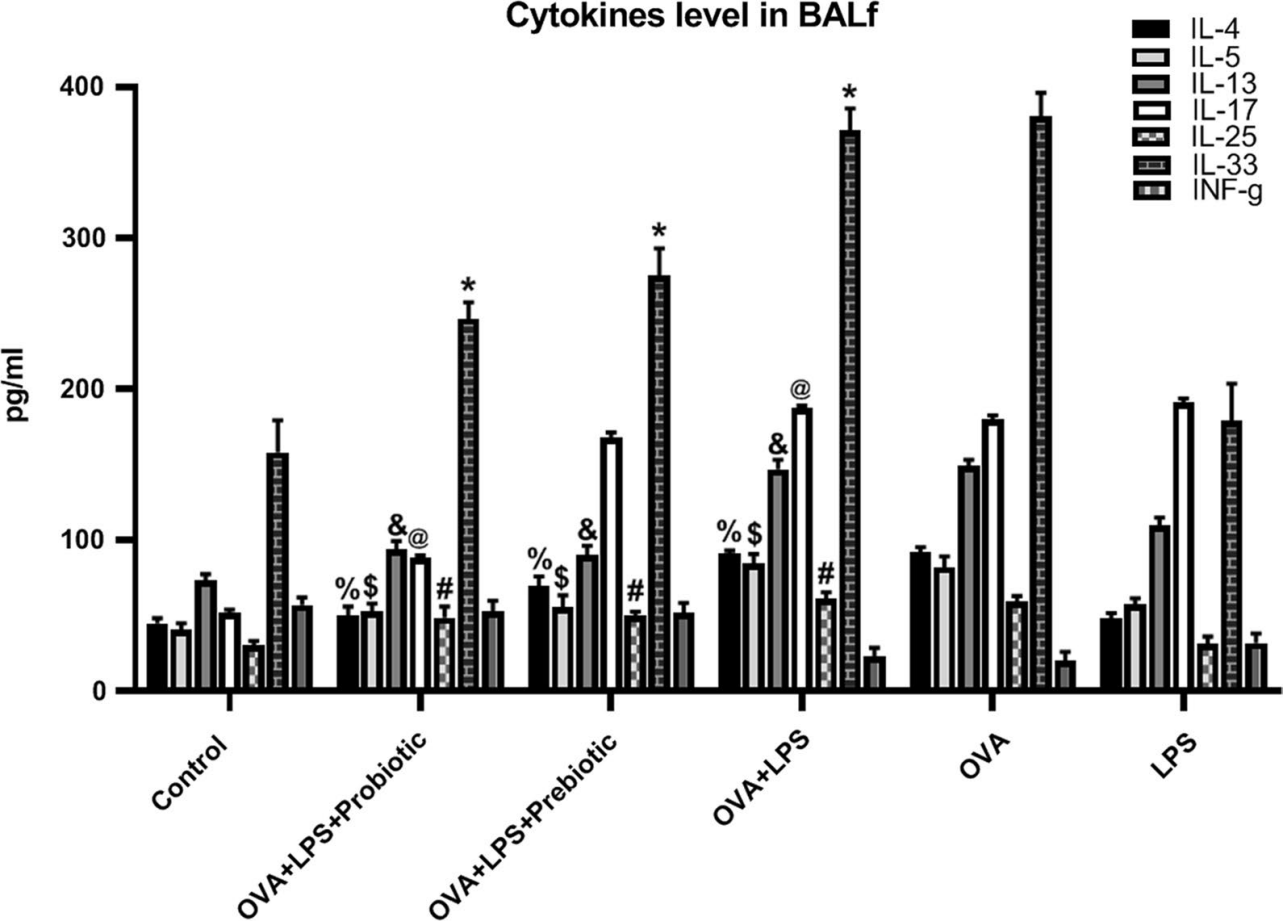
Cytokines. The levels of cytokines (IL-4, 5, 13, 17, 25, 33, and IFN-γ) were measured in BALF in all groups. The levels of IL-4, 5, 13, 17, 25, and 33 were increased significantly (*p* < 0.05) after sensitization/challenge with OVA and also OVA-LPS as compared to their levels in non-sensitized animals and the level of INF-γ were decreased in sensitized/challenged group. The pro- and pre-biotic treated reduced the levels of IL-4, IL-5, IL-13, IL-25 and IL-33 as compared to non-treated OVA-LPS induced asthmatic mice. The level of IL-17 was significantly decreased only in probiotics treated group. The levels of IFN-γ considerably (*p* < 0.05) increased in the probiotics and prebiotics treated groups. The significant difference (p < 0.05) between treated groups and non-treated OVA-LPS group was shown with symbols that include: * for IL-33, # for IL-25, @ for IL-17, & for IL-13, $ for IL-5, and % for IL-4

The effects of probiotics and prebiotics on Th2-related inflammatory and pro-inflammatory cytokines (IL-4, IL-5, IL-13, IL-17, IL-25, and IL-33) in BALF were analyzed by ELISA. The levels of all the cytokines analyzed in our study increased significantly (p < 0.05) after sensitization/challenge with OVA and also OVA-LPS as compared to their levels in normal control animals. Substantial reductions (p < 0.05) were observed in the BALF of pro- and pre-biotic treated animals regarding the levels of IL-4 (50.4 ± 5.7 and 69.7 ± 6.4 pg/ml, respectively), IL-5 (53.2 ± 4.9 and 55.7 ± 7.8 pg/ml, respectively), IL-13 (94.25 ± 5.24 and 90.25 ± 6.12 pg/ml, respectively), IL-17 (88.14 ± 2.07 and 167.9 ± 3.24 pg/ml, respectively), IL-25 (48.95 ± 7.24 and 50.24 ± 2.52 pg/ml, respectively), and IL-33 (246.36 ± 10.98 and 274.85 ± 18.25 pg/ml, respectively) as compared to OVA-LPS induced asthmatic mice (IL-4: 91.19 ± 2.03, IL-5: 84.84 ± 6.11, IL-13: 146.78 ± 6.25, IL-17: 187.02 ± 1.89, IL-25: 61.36 ± 4.25, and IL-33: 371.36 ± 14.36 pg/ml) (Fig. 1). The level of IL-17 was significantly decreased only in probiotics treated group, and treatment with prebiotics had no significant (p > 0.05) effect on decreasing of IL-17 level. Besides the induction of Th2-type inflammatory cytokines, allergen sensitization and provocation also significantly decreased IFN-γ level, a Th1-type cytokine, in the BALF. The levels of IFN-γ considerably (p < 0.05) increased in the animals treated with either probiotics or prebiotics (52.90 ± 7.16 and 51.74 ± 6.70 pg/ml, respectively) compared to the non-treated asthma group (22.85 ± 5.90 pg/ml), demonstrating that both the prophylactic treatments had the ability to modulate immune reactions by balancing Th1/Th2 responses in lungs (Fig. 1).

### ***Effect of probiotic and prebiotic treatments on total IgE, OVA-specific IgE, and IgG***_1_

**Fig. 2:**
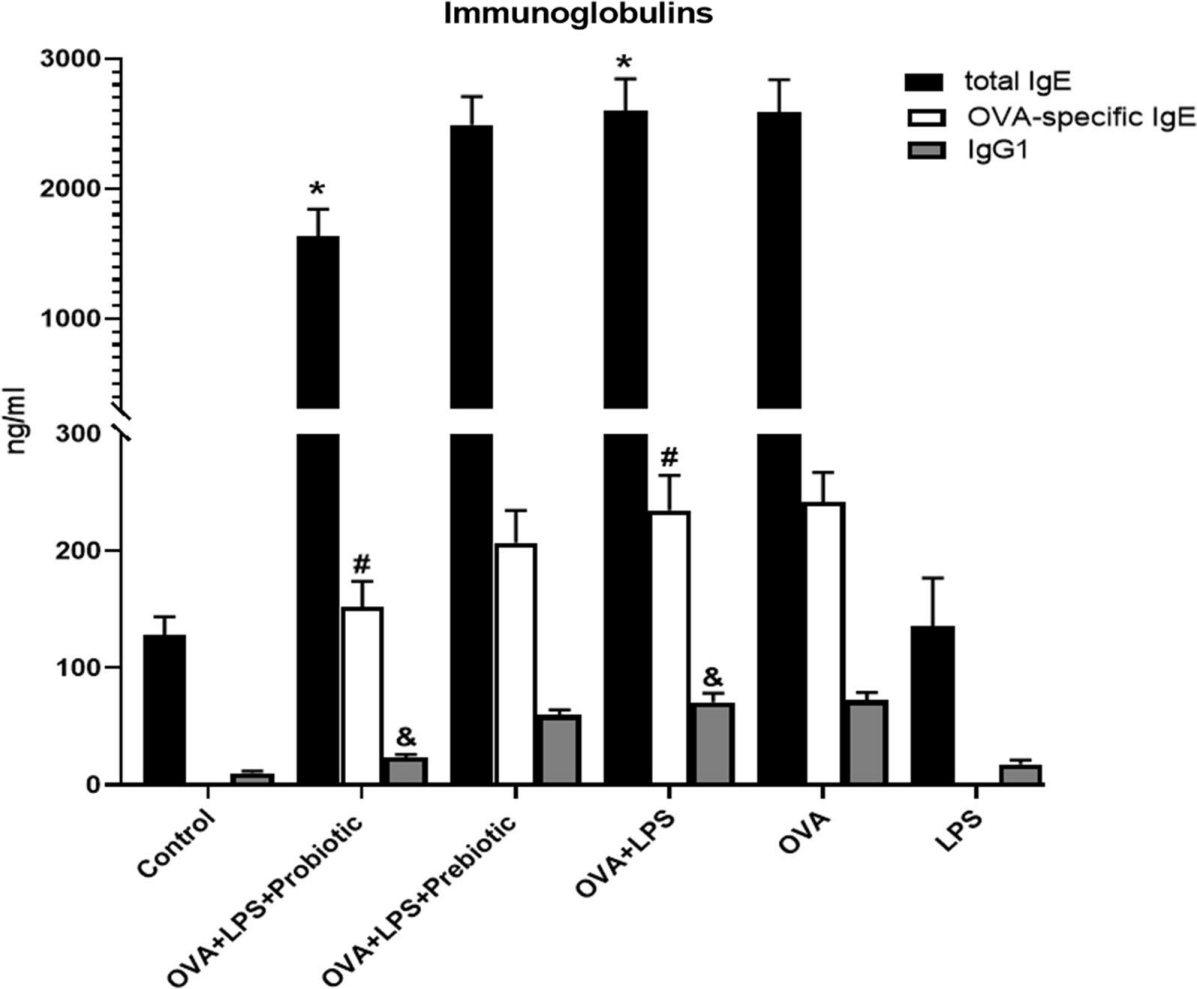
Immunoglobulins. The levels of immunoglobulins (total IgE, OVA specific IgE, and IgG1) were measured in all groups. The total IgE, OVA-specific IgE, and IgG_1_, were significantly increased in the OVA-LPS-sensitized mice compared to the healthy mice (*p* < 0.05). The administration of probiotics and prebiotics decreased the production of these immunoglobulins, but only probiotic treatment significantly decreased the production of total IgE, OVA-specific IgE and IgG1 (*p* < 0.05) compared to non-treated asthma group. The significant difference (p < 0.05) between treated groups and non-treated OVA-LPS group was shown with symbols that include: * for total IgE, # for OVA-specific IgE and & for IgG1

We next investigated the effects of probiotic and prebiotic treatments on the production of total IgE, OVA-specific IgE, and IgG_1_, showing that these immunoglobulins significantly (p < 0.05) increased in the sensitization group compared to the control group (healthy mice). The oral administration of both probiotics and prebiotics decreased the production of these immunoglobulins in OVA-LPS-sensitized mice as compared to untreated asthmatic animals (Fig. 2). Probiotic treatment significantly decreased the production of total IgE (p < 0.05), OVA-specific IgE (p < 0.05), and IgG1 (p < 0.05) compared to asthma control animals (group II). Treatment with prebiotics could decrease the levels of these immunoglobulins (total IgE, OVA-specific IgE, and IgG1), but the results were not significantly (p > 0.05) different compared to that of the asthmatic group (Fig. 2).

### Effect of probiotic and prebiotic treatments on leukotrienes’ levels

The levels of Cys-LTs, LTC4, and LTB4 were increased in OVA + LPS group (807.9 ± 37.3, 248.9 ± 20.1, 109.4 ± 9.3 pg/ml respectively) compared with control group (101.9 ± 4.7, 72.0 ± 28.0, 64.1 ± 7.1 pg/ml respectively). Assessing the effects of probiotics and prebiotics on the BALF levels of leukotrienes in asthmatic mice, we observed that the oral administration of probiotics significantly (p < 0.05) reduced the levels of Cys-LTs (374.9 ± 29.1 pg/ml), LTC4 (137.4 ± 24.8 pg/ml), and LTB4 (70.3 ± 9.1 pg/ml). Similarly, the levels of all the assessed leukotrienes decreased in the asthmatic animals treated by prebiotics (Cys-LTs: 429.7 ± 50.8, LTC4: 153.6 ± 37.9: and LTB4 (78.4 ± 7.8 pg/ml).

### Effect of probiotic and prebiotic on TSLP

Exposure to airborne allergens including fungal and viral pathogens elevates TSLP level in the epithelial cells of asthmatic lungs. In our study, OVA-LPS challenge in mice increased TSLP (2.8 ± 0.3 ng/ml) level in the BALF as compared to the normal control (0.4 ± 0.1 ng/ml). However, treatment with probiotics (2.1 ± 0.5 ng/ml) and prebiotics (2.6 ± 0.2 ng/ml) reduced TSLP level in the BALF as compared to asthma control animals, but these were not statistically significant (p > 0.05).

### Effect of probiotic and prebiotic treatments on GTP and GOT

Oxidative stress in the lung was examined using the glutathione assay. In our study, OVA-LPS challenge resulted in an increase in the levels of GTP (129.4 ± 21.4 IU/L) and GOT (419.7 ± 43.7 IU/L) as compared to the normal control group (GTP: 43.2 ± 10.3 and GOT: 119.4 ± 27.6 IU/L). Only, treatment with probiotics (64.5 ± 19.2 IU/L), but not prebiotics (101.6 ± 17.7 IU/L), significantly (p > 0.05) reduced GTP levels as compared to asthma control animals.

### Effect of probiotic and prebiotic treatments on the genes expressions

**Fig. 3:**
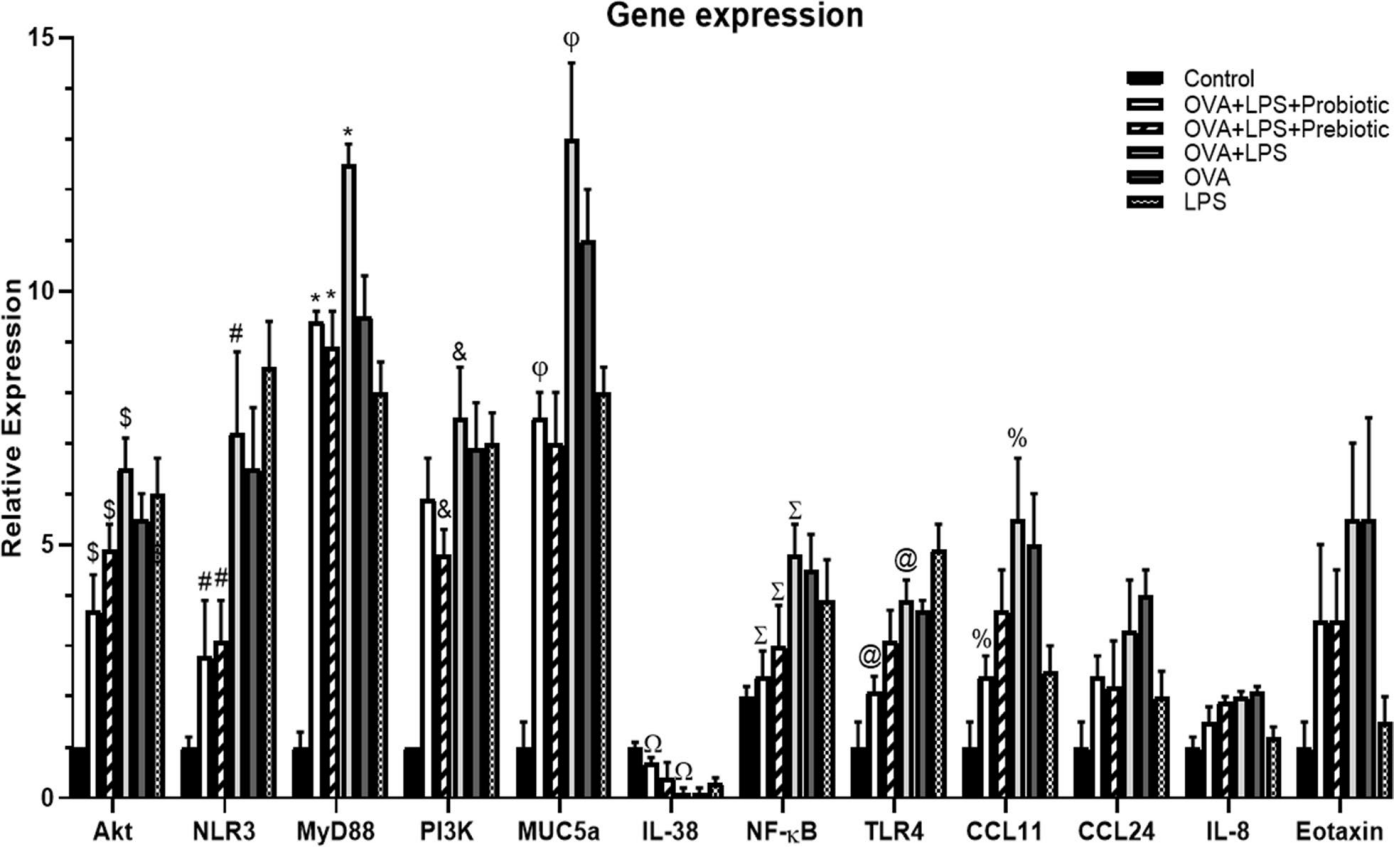
Gene expression. The gene expressions of Akt, PI3k, NLR3, MyD88, NF-κB, TLR4, CCL24, CCL11, Eotaxin, MUC5a, IL-8, and IL-38 were assessed in all groups. GAPDH was used as reference gene. It was observed that the expression levels of Akt, PI3K, NF-kB, and TLR4 significantly (p < 0.05) increased in the OVA-LPS group. The administration of probiotics and prebiotics dramatically attenuated the expression of Akt. Administration of probiotics and prebiotics remarkably inhibited the gene expressions of NF‐κB as compared to the asthma control group (p < 0.05). Treatment with probiotics could control TLR4 expression and prebiotics remarkably inhibited PI3K expressions. In our study, MyD88, MUC5a and NLR3 were up-regulated in the airways of OVA-LPS mice. However, MyD88, NLR3 and MUC5a expression significantly reduced (p < 0.05) in the animals treated with pro-/pre-biotics. The eotaxin gene expression increased in OVA-LPS sensitization/challenge animals. After treatment with probiotics and prebiotics; however, the level of eotaxin reduced (but it was not statistically significant; p > 0.05). On the other hand, the expressions of CCL11 and CCL24 were reduced in the pro- and pre-biotics treated mice, while there was a significant reduction in only CCL11 in the probiotics treated mice (p < 0.05). Also, the up-regulation of IL-8 gene was considerably inhibited following the treatment of OVA-LPS-sensitized mice with probiotics (but not statistically significant; p > 0.05). Allergen exposure significantly (p < 0.05) disturbed the expression of IL-38. Probiotics and prebiotics treatment increased IL-38 gene expression, but only probiotics treatment had significant effect (p < 0.05). The significant difference (p < 0.05) between treated groups and non-treated OVA-LPS group was shown with symbols that include: * for MyD88, # for NLR3, & for PI3k, @ for TLR4, $ for Akt, % for CCL11, Σ for NF-κB, Ω for IL-38 and φ for MUC5a

To evaluate the potential mechanisms of probiotic and prebiotic treatments in reversing the symptoms of asthma and inflammation in allergen-sensitized and challenged animals, the expressions of the genes involved in the PI3K/Akt and TLR4/NF‐κB signaling pathways were examined. The PI3K/AKT signal pathway contributes to the epithelial-mesenchymal transition and airway structural changes and remodeling in chronically and acutely inflamed asthmatic lungs. In an induced asthma model, the gene expression of Th-related cytokines and signaling molecules were assessed. Consistent with previous records, we also observed that the expression levels of Akt, PI3K, NF-kB, and TLR4 significantly increased in the non-treated asthma group. The administration of probiotics and prebiotics dramatically attenuated the expression of Akt. The gene expression of TLR4 and NF‐κB were also found to be elevated in asthmatic mice. As expected, the oral administration of probiotics and prebiotics remarkably inhibited the gene expression of NF‐κB as compared to the non-treated asthma group (Fig. 3). Treatment with probiotics could control expression of TLR4 and treatment with prebiotics remarkably inhibited expressions of PI3K. Taken together, probiotics and prebiotics seemed to be involved in regulating PI3K/Akt- and TLR4/NF‐κB-mediated immune responses.

The sensitization and provocation of mice airways with OVA and LPS are mediated by the MyD88. In our study, MyD88 was up-regulated in the airways of OVA-LPS-treated mice. However, MyD88 expression significantly reduced in the animals treated with pro-/pre-biotics. Similar results were observed for NLR3 (Fig. 3). In the mice models of asthma, MyD88 expression was found to be a critical factor for eosinophilic inflammation in ECs. The reduced expression of MyD88 after treatment with pro-/pre-biotics implicated their role in preventing eosinophilic infiltration in asthma.

Eotaxin is a chemotactic cytokine/chemokine with potent effects on eosinophils. The expression of eotaxin gene increases in asthmatic lungs after allergen challenge, leading to the accumulation of eosinophils in airways and impaired pulmonary function. The results of our study showed an increased expression of eotaxin after OVA-LPS sensitization/challenge in the animals. After treatment with probiotics and prebiotics; however, the level of eotaxin reduced (but it was not statistically significant; p > 0.05), revealing the ability of the both compounds in normalizing the expression of eotaxin in asthmatic lungs. Due to their chemotactic properties for eosinophils, CCL11 and CCL24 chemokines have attracted a great attention in allergic inflammation. Eotaxin-1/CCL11 is important for early eosinophil recruitment to the airways of asthmatics. The number of the cells expressing eotaxin-1 mRNA significantly correlated with the number of eosinophils. In our study, the observed significant increases in the expressions of CCL11 and CCL24 were consistent with the literature. On the other hand, the expressions of CCL11 and CCL24 in the BALF reduced in the mice treated with probiotics and prebiotics while there was a significant reduction in only CCL11 in the OVA-LPS sensitized/challenged mice treated with probiotics (p < 0.05). The data showed that prophylactic probiotics and prebiotics could inhibit the infiltration of eosinophils into asthmatic lungs (Fig. 3).

IL-8 is produced by numerous cell types including monocytes, neutrophils, basophils, eosinophils, alveolar macrophages, bronchial epithelial cells, pulmonary microvascular endothelial cells, and airway smooth muscle cells. In our study, OVA + LPS sensitization and provocation increased IL-8 expression in the BALF samples of asthmatic mice compared to healthy animals. The up-regulation of IL-8 gene was considerably inhibited following the treatment of OVA-LPS-sensitized mice with probiotics as compared to asthma control animals (but not statistically significant; p > 0.05). On the other side, prebiotic treatment did not elicit any significant improvement in the expression of this cytokine (p > 0.05). IL-38, a newly discovered cytokine belonging to the IL-1 family, has been shown to be aberrantly expressed in allergic asthma and COPD in clinical and animal studies. The cytokine suppresses the asthma-induced production of CXCL8, suggesting an inhibitory role for IL-38 in regulating allergic inflammatory responses. Allergen exposure significantly disturbed the expression of IL-38 in mice. All the asthmatic mice treated with oral probiotics and prebiotics showed an increased expression of IL-38 gene in the BALf (Fig. 3). Probiotics and prebiotics treatment increased IL-38 gene expression, but only probiotics treatment had significant effect (p < 0.05).

Increased mucus production is associated with the increased expression of mucin subfamily genes. Mucins are large glycoproteins constituting the major components of the mucus layer. They are produced by goblet cells or submucosal glands in airway epithelia. Out of two principal mucus forming enzymes, MUC5a and MUC5b, the overproduction of MUC5a has a major contribution to airway obstruction. In our study, treating asthmatic mice with probiotics and prebiotics significantly (p < 0.05) reduced MUC5a gene expression when compared to non-treated asthmatic mice (OVA-LPS group) (Fig. 3).

### Effect of probiotic and prebiotic treatments on histopathological study

**Fig. 4:**
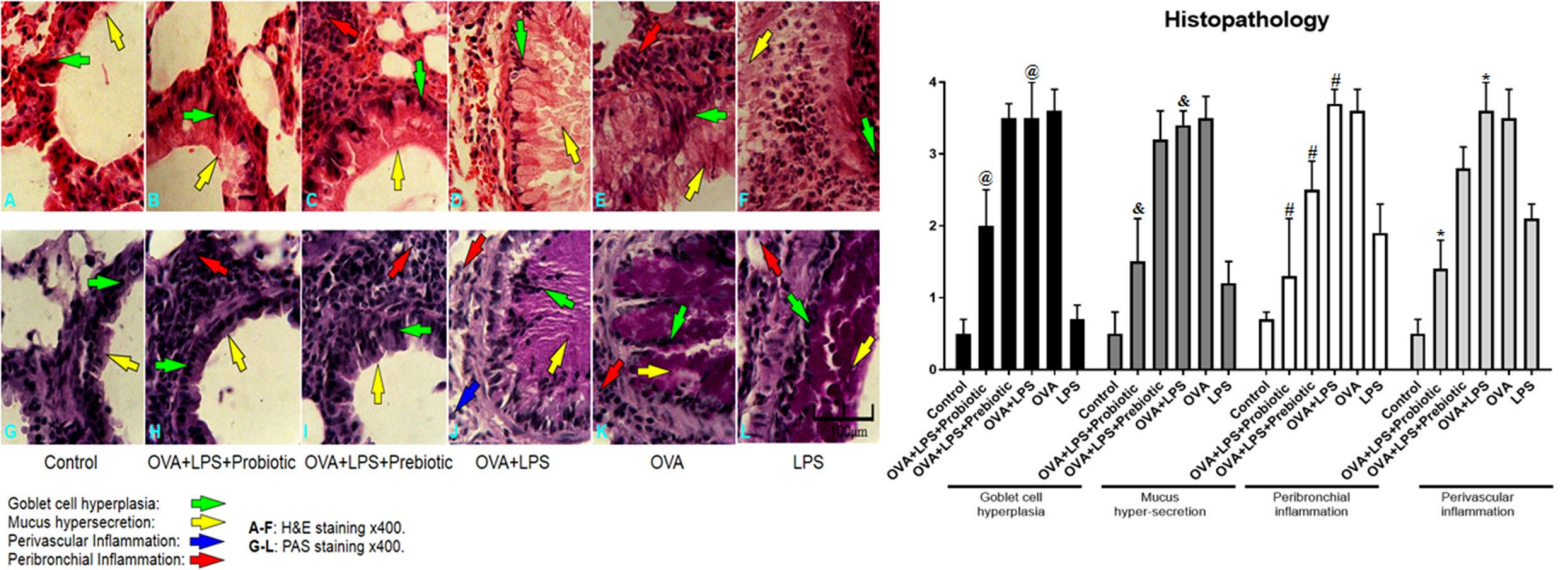
Lung histopathology. Lung sections were stained with H&E and PAS and the infiltration of inflammatory cells (eosinophils) into perivascular and peribronchial, along with goblet cell hyperplasia, and mucus hypersecretion were studied. The histopathological results showed that infiltration of inflammatory cells into the perivascular and peribronchial regions, goblet cell hyperplasia and mucus hyper-production were increased significantly (p < 0.05) in OVA-LPS-induced asthmatic mice compared with the PBS sensitized mice. A result analysis revealed a significant (p < 0.05) reduction in perivascular and peribronchial inflammation, goblet cell hyperplasia and mucus hyper-production in the probiotic-treated groups compared with the OVA-LPS group. Treatment with prebiotics could significantly (p < 0.05) control only peribronchial inflammation. The significant difference of the results (p < 0.05) between treated groups and non-treated OVA-LPS groups was shown with symbols that include: * for perivascular inflammation, # for peribronchial inflammation, & for mucus hypersecretion, and @ for goblet cell hyperplasia. In the histopathologic sections, hyperplasia of the goblet cells were shown by green arrow, hypersecretion of mucus was shown by yellow arrow, perivascular eosinophilc inflammation was shown by blue arrow and peribronchial eosinophilc inflammation was shown by red arrow

In order to assess the role of probiotics and prebiotics in reversing asthma symptoms in animal models, we examined the histopathological features of the lung tissue sections stained with H&E and PAS. In OVA-LPS-induced asthmatic mice, we observed marked pulmonary infiltration by inflammatory cells into the perivascular and peribronchial regions compared with the mice sensitized with PBS. A semi-quantitative analysis further revealed a significant reduction in inflammation in the probiotic-treated groups compared with the OVA-LPS group. The scores related to goblet cell hyperplasia and mucus production reduced significantly (p < 0.05) in the probiotic-treated group compared to OVA-LPS asthmatic animals. Treatment with prebiotics could control only peribronchial inflammation. On the other hand, we did not find any remarkable histological change in the animals treated with prebiotics (Fig. 4).

## Discussion

Atopy and allergy affect more than 30% of the global population. The prevalence of allergic diseases has been increasing in developed countries during the past 20 years and probiotics, as live microorganisms, have been beneficial for managing allergic diseases [5]. Clinical evidence showed the therapeutic role of probiotics in allergic rhinitis and asthma, improving cure rates and alleviating allergic rhinitis symptoms and the need for drug use. In clinical trials, receiving probiotics has led to the successful control of the immunologic outcomes of allergy (total and allergen specific IgE, leukocyte counts, blood and nasal eosinophils, Th2/Th1 ratio, IL-4 level, thymus-and activation-regulated chemokines, and quality of life). Also, probiotics increased the number of Treg and TGF-β-producing cells; on the other hand, Treg activity could modulate the severity of allergic inflammation [5, 6]. In general, probiotics can decrease disease severity mainly via modulating the immune responses involved in the development of allergic inflammation [5, 15]. According to hygiene hypothesis, in early childhood, lack of exposure to pathogens increases the susceptibility to allergic diseases such as asthma. It leads to Th1-like pattern immunity shits to the Th2-immunoallergic response. Probiotics in adequate amounts confer a health benefit to the host and have ability to modulate mucosal immune responses, stimulatory effects on the innate and adaptive immune system and enhance mucosal barrier functions [16, 17]. Moreover, the innate immune stimulation by bacteria is essential for the establishment of mucosal immune tolerance and, it can be continued by TLR2, TLR4, and TLR9 recognizing microbial components via production of Th1 cytokines. Probiotics may have a strong action in allergic pathologies via TLRs pathways. In particular, the probiotics play a pivotal role in the Th1/Th2 balance. In the allergic asthma, probiotics attenuate the influx of eosinophils to the airway and reduce the levels of MCP-1, and IL-13 level [16, 17]. We observed that probiotic and prebiotic treatments attenuated the recruitment of activated eosinophil to the lung and decreased EPO activity in BALF. Probiotic treatment also decreased eosinophils’ infiltration into BALF. The increased neutrophils in BALF of the LPS challenged groups was controlled by probiotics and prebiotics. LPS acts as TLR4 activator that in this study, increased and recruited neutrophils to the BALF and treatment with probiotics and prebiotics could markedly decrease neutrophils infiltration to the BALF. Therefore, TLR4 was involved in this study by LPS and effect of probiotic and prebiotic treatments on inflammation via TLR4 was noted. Probiotic and prebiotic treatments diminished the production of TSLP, cyst-LTs, LTC4, LTB4, GTP, and GOT which lead to control of inflammation in airways and harnessing of immune-inflammatory responses in lung and could control bronchial inflammo-pathology. Also, Probiotic and prebiotic treatments decreased the elevated levels of total IgE, OVA-specific IgE, and IgG_1_ and had effect on humoral immunity. The B cell activation is mainly controlled by T cells and T cell releasing cytokines. When B cells are activated can produce and release specific immunoglobulins. Allergen-activated B cells produce IgG_1_, total and OVA-specific IgE (in mice) and produced Igs bind to the specific receptors on surface of the mast cells, lead to mast cells degranulation and releasing of allegro-inflammatory mediators [2, 4]. In this study, treatment by probiotic had effect on T cell's cytokines and T cell response (cellular immunity) and may have directly effect on B cell to decrease of allegro-inflammatory immunoglobulins. Between two used treatment (probiotic and prebiotic), probiotic had stronger effect on control of allegro-inflammatory factors than prebiotic and could significantly decrease asthma related immune-inflammatory biomarkers. These results implicated that probiotics might have anti-inflammatory properties. In asthmatic lung tissues, the infiltration of inflammatory cells into perivascular and peribronchial areas and increased scores of goblet cell hyperplasia and mucus secretion were observed. This inflammation significantly decreased in probiotic- and prebiotic-treated animals. The scores of goblet cell hyperplasia and mucus secretion reduced in probiotic-treated mice.

On the other hand, according to the ‘hygiene hypothesis’, the increasing prevalence of immune-mediated anti-microbial activity may be related to the decreased incidence of allergic diseases. The crosstalk between gut microflora (probiotics) and the immune system (gut-associated lymphoid tissues) enables balancing Th1/Th2 immune responses in healthy individuals and mediates tolerance and mucosal immunity [6, 15, 17]. The effects of probiotics and prebiotics on Th2-related pro-inflammatory cytokines (IL-4, IL-5, IL-13, IL-25, and IL-33), Th1-related pro-inflammatory cytokine (IFN-γ) and Th17-related cytokine (IL-17) were analyzed. Probiotic and prebiotic treatments reduced the levels of Th2 cytokines (IL-4, IL-5, IL-13, IL-17, IL-25, and IL-33) and increased IFN-γ level in the BALF of asthmatic mice as compared to the non-treated asthma group. Also, gene expression of IL-8 was decreased, and IL-38 was increased in the probiotic- and prebiotic-treated groups compared with the non-treated asthma group, indicating the regulatory effects of probiotics and prebiotics on the Th1/Th2 balance. In this study, probiotic had surprising effect on modulation of cytokines levels in treatment of asthma, but prebiotic treatment had weak effect especially on cytokines levels and genes expression. It was reported that bacterial probiotics (*Bifidobacterium infantis*) treatment decreased levels of OVA specific-IgE, and IgG in serum, reduced the release of IL-4, -5, -13 in splenocytes. Moreover, the protective effect of probiotics on allergic asthma was correlated with superoxide dismutase and antioxidative enzyme [19, 20]. Treg cells have important role in asthma, and less functional Treg cells lead to enhanced Th2-type immune responses, and eosinophilic airways inflammation. High-fibre prebiotics, via the acetate production, prime Treg cell to protect against asthma. Prebiotics such as soluble fibres and inulin should be fermented by beneficial bacteria and this is time-taking process. Therefore, these products cannot quickly act and present anti-inflammatory properties [13, 21].

Several studies showed that prebiotic supplementation improved airway hyperresponsiveness and decreased inflammatory cells’ counts in the sputum of asthmatic patients [13, 22–24]. Penh is a dimensionless index that generally evaluates changes in the shape of the airflow pattern entering and leaving a whole-body flow plethysmograph when an animal breathes and also by monitoring of AHR changes via connected tube to the ventilator. A higher level of Penh indicates a more severe bronchospasm/airway hyperreactivity to allergens. Interestingly, the oral administration of probiotics and prebiotics could improve AHR. Our results revealed that probiotic administration, but not prebiotic, significantly decreased AHR in asthmatic animals. Nevertheless, prebiotic treatment also reduced AHR, but the results were not significant that was similar to a study in 2020 [25].

LPS as part of the cell membrane of Gram-negative bacteria is one of TLR4 ligands. When LPS binds to TLR4, activates the MyD88 leading to the downstream production of kinases, which allow translocation of the NFκB-related proteins into the nucleus. The NF-κB complex triggers transcription of genes that are involved in the immune-inflammatory responses, and once activated begins a cascade of pro-inflammatory cytokine, specifically TNF-α, IL-1β and IL-6. Once activated NLRP3 inflammasome via microbial components cleaves pro-IL-1β, -IL-18, leukotrienes and prostaglandins [26–28]. In this study, LPS was used as TLR4 activating factor for airway inflammation. When TLR4 in involved, it can participate in asthma pathophysiology and treatment with probiotic has influence on the TLR4 and related signaling pathways. Therefore, the effect of probiotic on regulation of TLR4/ NF-kB Signalling Pathway will be notable. In this study, it was observed that probiotic therapy could decrease and control gene expression of TLR4, NLRP3 and NF-kB as main inflammatory molecules.

It has been demonstrated that many factors and signaling molecules contribute to the induction of allergic inflammation, including cytokines, chemokines, superoxide, ECP, TLRs, and intracellular kinases (NF-κB, ERK, Akt, p38, MAPK, etc.) [3, 29, 30]. AngII could increase ROS production and oxidative stress by activating NADPH oxidases, and promoting release of inflammatory cytokines. It was demonstrated that AngII exhibits pro-inflammatory responses via TLR4 upregulation and stimulating TLR4-dependent signaling pathways. Likewise, AngII upregulates TLR4 and MyD88 gene and AngII-TLR4 crosstalk promotes pro-inflammatory effects, and also, AngII-induced ROS production is dependent on the presence of a functional TLR4. AngII-induced TLR4 upregulation promotes functional consequences such as increase in NF-kB activation, which is dependent the presence of functional AP-1, ETS, and PU.1 transcriptional site. Moreover, molecular mechanisms of AngII-TLR4 activation may indicate an interaction between TLR4 and AT1r signaling downstream effector molecules. Also, TLR4-mediated NF-kB activation in response to LPS is dependent on EGFR signaling. These suggest a regulatory mechanism between AT1r, TLR4, and NF-kB indicating their role in pro-inflammatory effects [31, 32]. Moreover, AngII can directly activate the NF-κB that is critical for initiating the inflammatory cytokines responses during the inflammation. Also, during the inflammatory process, activation of the TLR4 could induce the pro-inflammatory cytokines production and activation of NF-κB via MAPK signaling pathway. On the other hand, TLR4 expression is modulated by local AngII and caused the activation of NF-κB signaling and induction of TNF-α, CD40, and IL-6 expression (33). In this regard, probiotics were used in this study to modulate these pathways. Probiotic and prebiotic treatments reduced the expression of asthma related genes involved in the PI3K/Akt and TLR4/NF‐κB signaling pathways in asthmatic lungs. As we observed, the expression levels of Akt, PI3K, NF-kB, and TLR4 raised in asthmatic mice, and the administration of probiotics dramatically attenuated the expression levels of these genes. The oral administration of probiotics and prebiotics remarkably inhibited the gene expressions of TLR4, NLR3, NF‐κB, and MyD88, but decreasing of TLR4 gene expression by prebiotics was not significant.

IL-4 induces GATA3 expression and in result Th2 cell differentiation. OX40 ligand is linked to the development of Th2-related allergic responses. The activity of probiotic (Lactobacillus murinus) against food allergy showed that oral administration attenuated allergen-induced diarrhoea, mast cell activation, serum IgE and IL-4 production by splenocytes. It enhanced IFN-γ and IL-12 production. Concordantly, it suppressed expression GATA3 and OX40 ligand expression by intestinal CD11c + cells and increased T-bet expression. These demonstrate that probiotic possesses anti-allergic activities via modulating CD11c + cell functionality and the Th1/Th2 immunobalance, which may be employed against food allergy [34, 35]. IL-38 acts as a partial antagonist receptor of IL-36 inflammatory cytokine, suppressing the asthma-induced production of CXCL8 and inhibiting allergic inflammatory responses [36–38]. IL-38 may regulate airway inflammation and can diminish the production of the inflammatory cytokines and chemokines that are regulated by the intracellular p38, ERK1/2, and NF-κB signaling pathways [39, 40]. Eotaxin, a chemotactic cytokine/chemokine with potent effects on eosinophils, increases in asthmatic lungs after exposure to allergens, causing the accumulation of eosinophils in airways. After treatment with both probiotics and prebiotics, the gene expression of eotaxin, CCL11, and CCL24 reduced in the lungs of asthmatic mice, but only CCL11 gene expression by probiotic was significant.

Bronchial inflammation is principal problem in asthma and free oxygen radicals have main role in airway inflammation and pathogenesis of allergic asthma. The oxidation and antioxidative defense modulation is new therapy in management of asthma. Mitochondrial abnormalities have collaboration with oxidative stress that can cause airways injury. Co-Q10 is one of the important antioxidant supplementations, which contributes in the regulation of mitochondrial function and pathology, and also, allergic inflammation. CO-Q10 can induce resistance against the asthma development, protect upper and lower airways against allergic response, and control inflammation [32, 41]. The effects of probiotics on the immune system are classified into two categories involving the activation of the innate immune cells and the inhibition of excessive abnormal immune responses [42, 43]. Oxidative stress is involved in the pathogenesis of several tissue damages and expression of striatal proteins contribute to oxidative stress and damage. ROS production and subsequent toxic events including; mitochondrial membrane potential collapse, lipid peroxidation, lysosomal membrane injury and enzymes dysfunction indicating that oxidative stress. Mitochondrial ROS formation is important mediator in tissue damage and antioxidants combat with free radicals to control oxidative stress. Also, angiotensin II is produced by ACE and can bind cell-surface receptors via AT1 and type 2. AT1 receptor is linked to ROS formation, and inflammation via activation of several downstream signals including, Ras/Rho, ERK, MAPK and JAK/STAT signaling pathway [44, 45]. Probiotics and prebiotics can shift immune reactions from Th2 to Th1 responses in allergic diseases, promoting tolerance in allegro-inflammatory conditions. Probiotics also modulate cellular (Th1, Th2,) and humoral (B cells) immune responses and improve general health by preventing allergies and immune disorders. Probiotic could reduce oxidative and inflammation markers and control airway inflammation and asthma pathophysiology, but prebiotic could not strongly control the inflammation and allergic asthma symptoms. Probiotics use major mechanisms to improve allergic asthma clinical symptoms and prevent them, with several known mechanisms: Th2 suppression and shift response to Th1, tolerance induction, increase of IL-10, Treg cells and their responses for control immune-inflammation, increase the IFN-γ/IL-4 ratio, decrease eosinophil, Th2 cytokines and IgE levels, reduce gene expression of allegro-immune response related, chemokines and cytokines signaling pathways, and also, control AHR. Finally, probiotics can be used as therapeutic approach to treatment of allergic asthma with priority of the tolerance and the lowest side effects.

The probiotics act through several mechanisms to maintain the homeostasis and perform beneficial functions, including pathogens elimination, vitamins and short-chain fatty acids production, as well as modulation of the systemic and mucosal immune system and also inflammation via controlling of NF-κB activation and pro-inflammatory cytokines. Probiotics are live organisms can act as delivery systems and proliferate in tissues for long time, but prebiotics have not these characters. Also, mechanisms action of probiotics in the host include; colonization, regulation of a dysbiotic microbiota, epithelial barrier protection by maintaining tight junction integrity, competitive exclusion of pathogens, production of anti-inflammatory properties, signaling molecules regulation of immunological pathways and inducing host autophagy to attenuate oxidative stress-induced injury. Prebiotics may be added as supplementary agent and stimulate probiotic action. Prebiotic fermentation can modulate the composition and function of probiotics. Prebiotics with regulatory ability, modify the commensal microbiota to a beneficial state. Prebiotics, when associated with a probiotic, are more efficient than when are used separately (13, 14, 46). Therefore, prebiotics may show lower effect than probiotics on control of eosinophilic airway inflammation, EPO activity, immune-allergic response, and allergic asthma.

In this study, there were some limitations. We did not study the effect of probiotic and prebiotic on control group and also on the OVA group and LPS group. We did not have data about protein of the signaling pathways, and phosphorylation of pathway proteins. We could not study to verify the pathway using genetic gain/loss of function models or pharmacologic treatments. We could not show that probiotics can inhibit asthma pathophysiology in absence of TLR4. Also, we did not study the effect of probiotic and prebiotic on chronic form of airway inflammation and asthma.

## Data Availability

Not applicable.

## Abbreviations

ACE: Angiotensin converting enzyme
AngII: Angiotensin II
AHR: Airway hyperresponsiveness
Akt: Protein kinase B
AP-1: Activator protein 1
AT1: Angiotensin II receptor type 1
BALF: Bronchoalveolar lavage fluid
BW: Body weight
CCL: Chemokine (C–C motif) ligand
CD: Cluster of differentiation
CFU: Colony forming units
COPD: Chronic obstructive pulmonary disease
CXCL: C–X–C Motif chemokine ligand
Cys-LT: Cysteinyl Leukotriene
EGFR: Epidermal growth factor receptor
ELISA: Enzyme-linked immunosorbent assay
Eos: Eosinophil
EPO: Eosinophil peroxidase
ERK: Extracellular signal-regulated kinases
ETS: Erythroblast Transformation Specific
FOS: Fructo-oligosaccharides
FOXP3: Forkhead box P3
GCI: Goblet Cell Index
GOS: Galacto-oligosaccharides
GOT: Glutamic oxaloacetic transaminase
GPR: G protein-coupled receptor
GTP: Glutamic pyruvic transaminase
HDAC: Histone deacetylase
H&E: Haemotoxylin and eosin stain
IFN: Interferon
Ig: Immunoglobulin
IL: Interleukin
IN: Intranasally administration
IP: Intraperitoneal injection
JAK: Janus Kinase
LPS: Lipopolysaccharide
LT: Leukotriene
MAMP: Microbe-associated molecular pattern
MAPK: Mitogen-Activated Protein Kinases
MCh: Methacholine
MCP: Monocyte chemoattractant protein
MUC5a: Mucin 5a
MyD88: Myeloid differentiation primary response 88
NF-kB: Nuclear factor kappa-light-chain-enhancer of activated B cells
NK: Natural killer cells
NLR: NLR Family Pyrin domain containing
OVA: Ovalbumin
OX40: Tumor necrosis factor receptor superfamily member 4 (TNFRSF4)/or CD134
RNS: Reactive nitrogen species
ROS: Reactive oxygen species
PAMP: Pathogen-associated molecular pattern
PAS: Periodic-acid-Schiff stain
PBS: Phosphate-buffered saline
PI3K: Phosphoinositide 3-kinase
RLR: Retinoic acid-inducible gene-I (RIG-)-like receptor
PO: Per oral
PRR: Pattern recognition receptor
STAT: Signal Transducer and Activator of Transcription
TGF: Transforming growth factor
Th: T helper
TLR: Toll-like receptor
TNF: Tumor necrosis factor
Treg: Regulatory T cell
TSLP: Thymic stromal lymphopoietin
